# On the danger of detecting network states in white noise

**DOI:** 10.3389/fncom.2015.00011

**Published:** 2015-02-12

**Authors:** Jaroslav Hlinka, Michal Hadrava

**Affiliations:** ^1^Department of Nonlinear Dynamics and Complex Systems, Institute of Computer Science, Academy of Sciences of the Czech RepublicPrague, Czech Republic; ^2^Department of Cybernetics, Faculty of Electrical Engineering, Czech Technical University in PraguePrague, Czech Republic

**Keywords:** EEG, microstates, networks, dynamics, resting-state, nonstationary connectivity, stationarity, white noise

The general idea of nonstationarity of brain activity or dependence of the dynamics on some, potentially unobserved, temporally changing or fluctuating parameter, has been familiar in the neuroscience community in contexts such as sleep dynamics or epileptology for a long time. However, recently it has been attracting increasing attention in the context of functional brain network analysis. This seems as a natural development of the field—once that functional connectivity as computed under the simplifying stationarity assumption has been well established, it is only logical to try to detect changes in brain functional connectivity over time. In general, detecting such nonstationarities in a reliable fashion is a methodologically challenging task, as changes in *estimates* of functional connectivity over time may be also due to random fluctuations, rather than genuine changes of the process. There is a wide array of approaches to studying such nonstationarities documented in literature (Hutchison et al., 2013), and an important but often neglected general methodological step is assessing the results against an appropriate null model corresponding to stationary process.

In the following, we give an illustrative example of how a typical nonstationarity analysis can generate spurious signs of nonstationary dynamics even when applied to stationary process. To show that this is not a purely theoretical issue, we closely follow the analysis procedure used in a recently published study by Betzel et al. (2012). We note that this particular paper have caught our attention by coincidence, while we believe the issue is pertinent to a substantial fraction of the literature.

In their paper, (Betzel et al., 2012) deal with characterizing the dynamics of brain activity measured by EEG. In particular, Betzel et al. report the detection of rapid transitions between intermittently stable states, explicitly saying that *“As predicted, fast (~100 ms) dynamics of whole-brain synchronization were observed during resting-state EEG,”* documenting the typical fast (~100 ms) time scale of these states in Figure 6B of their paper (see also Figures 4, 5). Their argument is based on the following data-processing scheme: First, for each time point of filtered EEG data, a functional connectivity matrix is computed using pairwise synchronization likelihood values and the time points are clustered based on similarity of the corresponding functional connectivity matrices. Next, contiguous stretches of time points that are members of the same cluster are interpreted as corresponding to a duration of an atomic brain state. Finally, the brain-state-representing functional connectivity matrices are pooled across subjects and clustered based on their similarity to define higher-order states.

Notably, the procedure applied by Betzel et al. is principally data-driven, rather than relying on some model testing or assumptions, and it includes band-pass filtering and sliding-window-like analysis. We therefore conjectured that the temporal structure of the observed functional connectivity dynamics might have been crucially affected by the procedure itself (as the authors tentatively admitted in their discussion, albeit unfortunately have not tested the results against stationary model data). To explore the viability of this alternative explanation, we applied a processing pipeline built according to the description given in the original manuscript to model data, consisting of 100 samples (each of length *T* = 2500 time points, representing mock 5 s epoch of EEG data) of a multivariate (*N* = 20) white noise process. The applied processing steps included application of frequency filtering (using elliptic filters corresponding to the four specified frequency bands; we applied zero-phase digital filtering by processing the input data in both the forward and reverse directions) and subsequent computation of the synchronization likelihood (Stam and van Dijk, 2002). The parameters of the synchronization likelihood *l, m, w*_1_, *w*_2_, *n_rec_* were set for each frequency band as in Betzel et al. (2012). The resulting functional connectivity matrices were clustered using the standard *k*-means clustering method (Lloyd, 1982). In Figure 1 you see that the typical duration of detected states closely corresponds to the distributions observed in the original paper (compare with Figures 6B, 4A,B in Betzel et al., 2012). In particular, the typical timescale is in the order of tens to hundreds of ms. Also, this time scale depends on the selected filtering in the same way as in the original work, with the time scales of the beta and theta bands markedly shorter and longer, respectively, than those of the broadband and alpha bands, the latter two being relatively close to each other.

**Figure 1:**
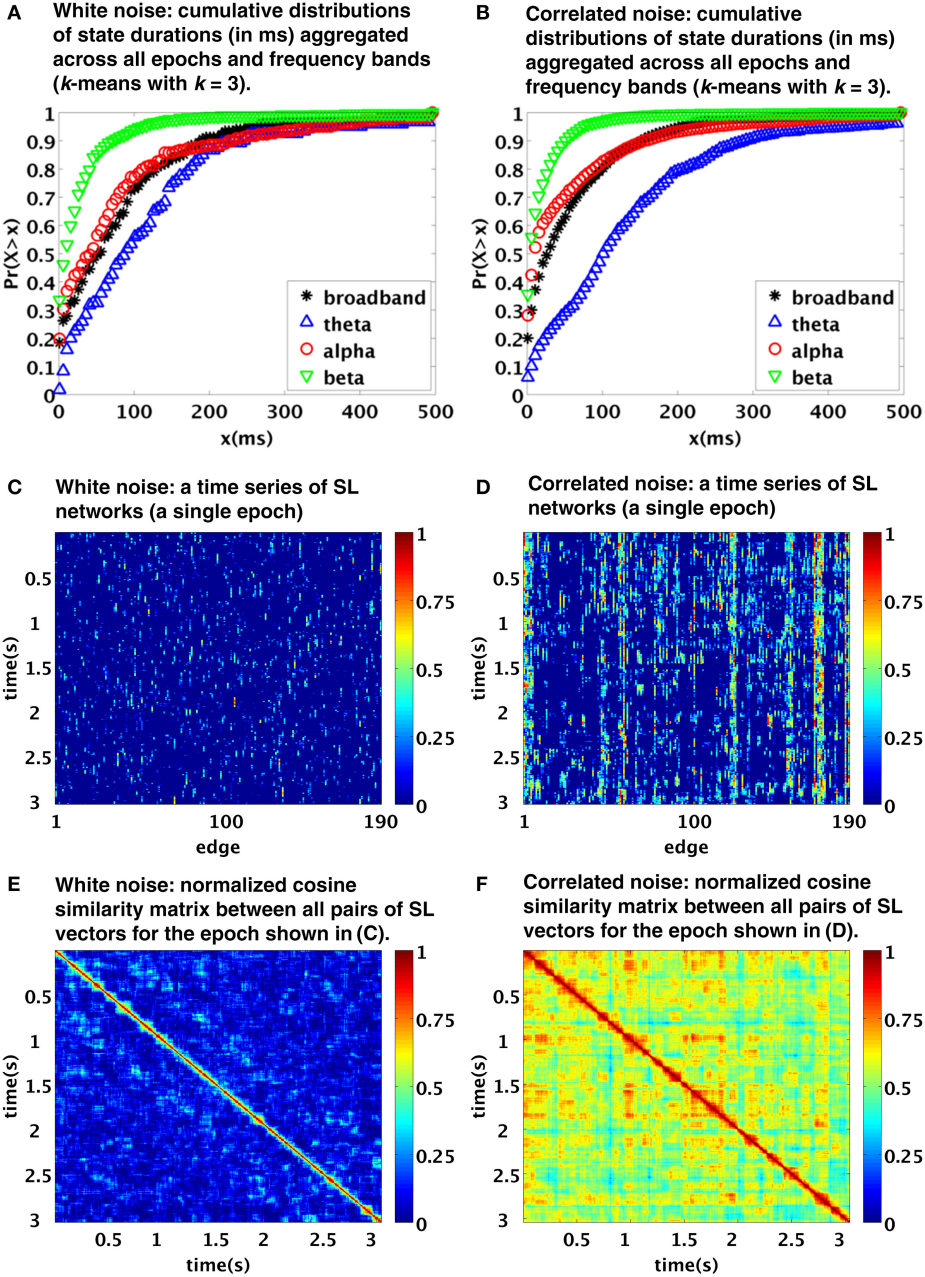
**Temporal dynamics of synchronization likelihood (SL) networks generated from realizations of stationary processes: white noise (A,C,E) and correlated noise [linear stationary (FFT) surrogates from EEG data] (B,D,F)**. The top, middle and bottom images were created using analogous procedures as Figures 6B, 4A,B of the original paper. Note the similarity to figures in the original paper, suggesting a possible role of processing pipeline rather than genuine “state switching” in the observed time scales.

Even though spatially and temporally independent (white) noise model used here is clearly not a realistic model for EEG data; such a simplistic stationary model reproduces the clustering time scales of the original paper with a surprising accuracy. Of course, due to spatial independence of the processes, it does not reproduce the spatiotemporal patterns corresponding to Figures 4A,B in Betzel et al. (2012). We have further repeated the procedure using multivariate Fourier transform surrogates generated from a single segment of EEG data (for more details on the data see Horacek et al., 2010. Such surrogates correspond to realizations of linear stationary process with conserved auto- and cross-correlation structure, see Prichard and Theiler, 1994). The results are shown in the right column of Figure 1. Moving from white noise to EEG surrogates, the time scales of the observed clustering hardly changed. However, as expected due to the introduced spatial dependence, the EEG surrogates show now a patchy spatiotemporal pattern Figures 1D,F more closely corresponding to those in the original paper. The similarity of the spatiotemporal patterns is of course only qualitative—range of differences may have arisen due to combination of different acquisition parameters as well as intra- and inter-individual variability.

Note that we applied the basic *k*-means clustering method instead of the evolutionary-clustering algorithm from the original paper; insufficient detail of description of the procedure in the original paper made reproducing it prohibitively difficult. The value *k* = 3 was chosen for display of the clustering results, however, the results proved to be quite insensitive to the choice of *k*.

Our numerical simulation above focused particularly on the observed *time scales* of the network states as obtained with the described analysis approach. One could indeed ask further, what evidence regarding “repertoire of states” can be provided by the detection of clusters *per se*—and whether the detection of (some) clusters could be merely a consequence of running a clustering algorithm. For a *k*-means clustering, the answer is obvious. Even for more complex approaches without fixed number of clusters such as the approach of Betzel et al. (2012), we conjecture that a repertoire could be observed even for a stationary process, however this depends on the details of applied analysis approach.

In summary, we aimed to illustrate the proposition that spurious nonstationarity manifesting itself as alternation of network states may appear due to methodological issues even in stationary processes such as white noise. In our example, we showed that for instance the observation of clustering of time points (more precisely, temporal windows) into consecutive clusters (“states”) of duration in the order of several hundred milliseconds (the time scale of putative brain microstates) might be reproduced by white noise to a remarkable detail. Of course, this does not *disprove* the existence of such states—it just suggests the evidence may not be sufficient.

From a wider perspective, one could see a parallel here with other examples of data analysis approaches that may lead to spurious observation of intriguing structures due to intrinsic bias of the methods—such as apparent signs of chaos in power-law spectra stochastic processes (Osborne and Provenzale, 1989) or small-world properties of functional connectivity graphs (Hlinka et al., 2012). Or, from an experimental point of view, with the role measurement artifacts such those as due to head motion might play in observed network properties (Hlinka et al., 2010; van Dijk et al., 2012).

## Conflict of interest statement

The authors declare that the research was conducted in the absence of any commercial or financial relationships that could be construed as a potential conflict of interest.
